# Supplementary material for: Enterobacter asburiae E7, a Novel Potential Probiotic, Enhances Resistance to Aeromonas veronii Infection via Stimulating the Immune Response in Common Carp (Cyprinus carpio)
Source: Microbiol Spectr. 2023 Mar 28;11(2):e04273-22. doi: 10.1128/spectrum.04273-22 (PMC10100819; doi:10.1128/spectrum.04273-22)
Supplement: Supplemental file 1 — Supplemental material. Download spectrum.04273-22-s0001.pdf, PDF file, 0.4 MB [file spectrum.04273-22-s0001.pdf]

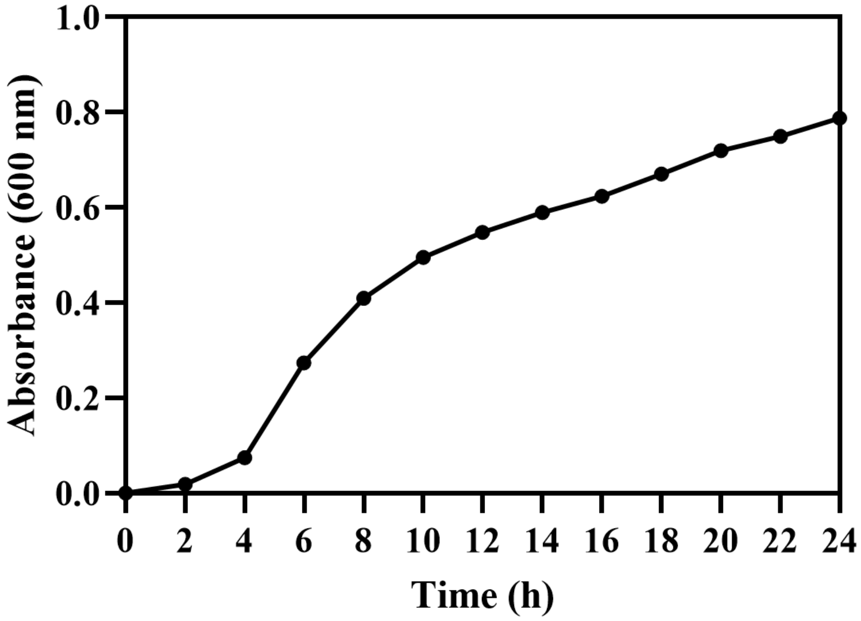

Supplementary Fig. 1. Growth curve of *E. asburiae* E7. Bacteria were inoculated in 100 mL LB broth medium (1%) at 160 rpm and 28℃. The absorbance at 600 nm was measured every 2 hours for 24 hours.

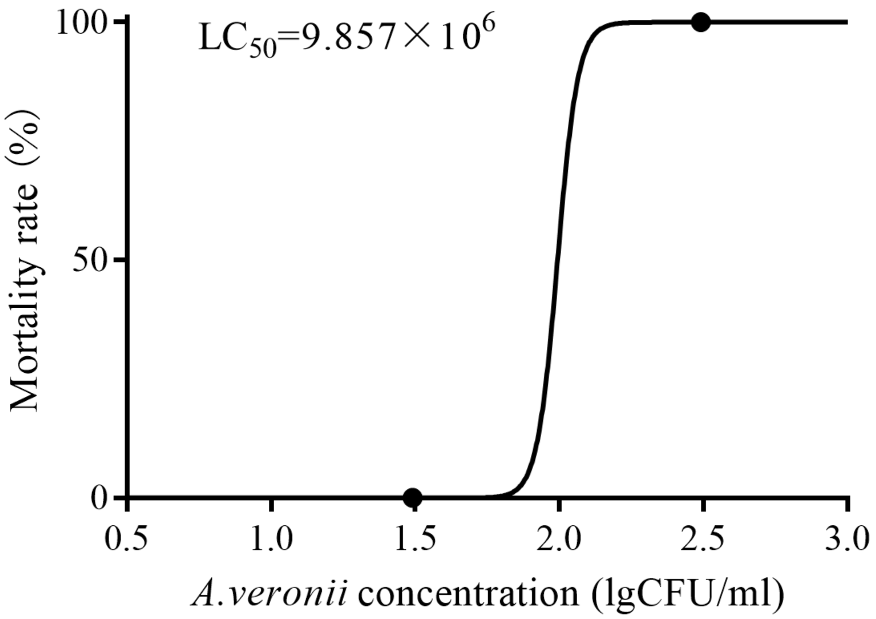

Supplementary Fig. 2. The toxicity of *A. veronii* on common carp. Common carp were ip injected with 0.1 mL *A. veronii* suspension with different concentrations (1×10^5, 1×10^6, 1×10^7, 1×10^8, 1×10^9 CFU/mL) for 7 days (n = 10).

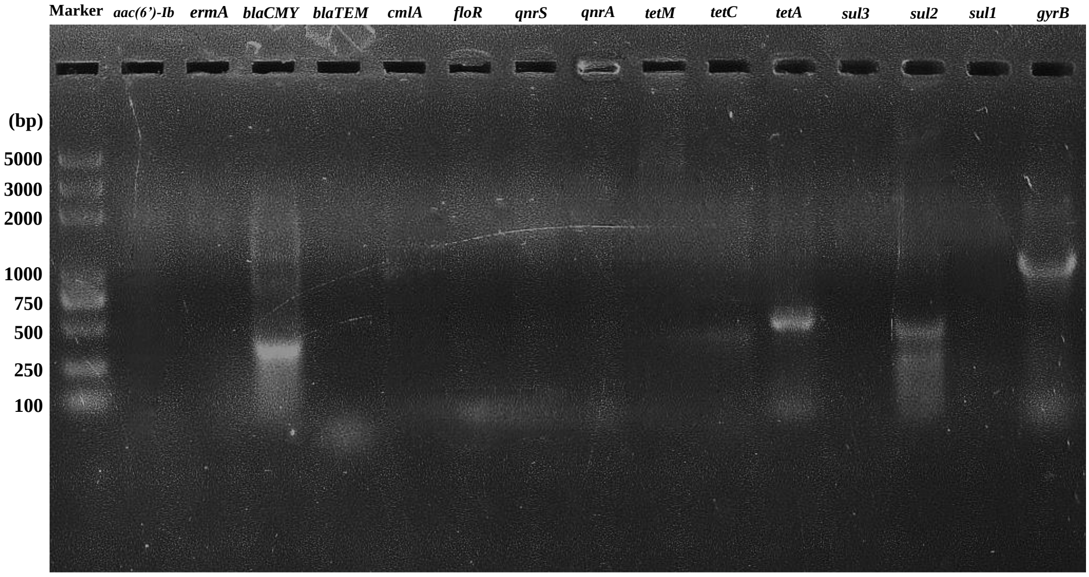

Supplementary Fig. 3. Antibiotic resistance genes (ARGs) of *E. asburiae* E7 were detected by PCR coupled with agarose gel electrophoresis. Lane 1: Trans 2K plus DNA Marker. Lanes 2-15: *aac(6')-Ib*, *ermA*, *blaCMY*, *blaTEM, cmlA*, *floR*, *qnrS*, *qnrA*, *tetM*, *tetC*, *tetA*, *sul3*, *sul2* and *sul1*. Lane 16, Positive test results of *gyrB* gene. The PCR product was analyzed by electrophoresis in 1.5% (w/v) agarose gel for 40 min at 100 V and 50 mA.

Supplementary Table 1

Primer sequences for antibiotic resistance genes (ARGs).

Gene name	Sequences of primers (5′to 3′)	Accession no.
sul1	F: CATTGCCTGGTTGCTTCAT<br>R: ATCCGACTCGCAGCATTT	AB061794
sul2	F: CATCATTTTCGGCATCGTC<br>R: TCTTGCGGTTTCTTTCAGC	NC005324
sul3	F: AGATGTGATTGATTTGGGAGC<br>R: TAGTTGTTTCTGGATTAGAGCCT	AY316203
tetA	F: GGCACCGAATGCGTATGAT<br>R: AAGCGAGCGGGTTGAGAG	X75761
tetC	F: CTGGGCTGCTTCCTAATGC<br>R: AGCTGTCCCTGATGGTCGT	J01749
tetM	F: GAGGTCCGTCTGAACTTTGCG<br>R: AGAAAGGATTTGGCGGCACT	DQ534550
qnrA	F: ATTTCTCACGCCAGGATTTG<br>R: GATCGGCAAAGGTTAGGTCA	DQ356006
qnrS	F: CATACATATCGGCACCACAAC<br>R: CAGGATAAACAACAATACCCAGT	EF683584
floR	F: GGCTTTCGTCATTGCGTCTC<br>R: ATCGGTAGGATGAAGGTGAGGA	AF261825
cmlA	F: TGCCAGCAGTGCCGTTTAT<br>R: CACCGCCCAAGCAGAAGTA	AJ487033
blaTEM	F: ATGAGTATTCAACATTTCCG<br>R: ACCAATGCTTAATCAGTGAG	NG050290
blaCMY	F: GCACTTAGCCACCTATACGGCAG<br>R: GCTTTTCAAGAATGCGCCAGG	MH426965
ermA	F: ATGCTTCAAAGCCTGTCGGA<br>R: GCGGTAAACCCCTCTGAGAA	X03216
aac(6')-Ib	F: ATGACCTTGCGATGCTCTATG<br>R: CGAATGCCTGGCGTGTTT	AJ009820
